# A Novel Preparation Method of Composite Bolted T-Joint with High Bending Performance Based on the Prepreg-RTM Co-Curing Process

**DOI:** 10.3390/polym16091259

**Published:** 2024-05-01

**Authors:** Tao Zhang, Zhitao Luo, Jinxin Deng, Yuchen Pei, Xiaoquan Cheng

**Affiliations:** 1 School of Materials Science and Engineering, Beihang University, Beijing 100191, China; 13911997771@139.com; 2 Research Institute of Aerospace Special Materials and Processing Technology, Beijing 100074, China; pei_yuchen@sina.com; 3 School of Aeronautic Science and Engineering, Beihang University, Beijing 100191, China; luo0327@buaa.edu.cn (Z.L.); hanxiebaoxin@buaa.edu.cn (J.D.)

**Keywords:** polymer composite, T-joint, preparation method, prepreg-RTM co-curing process, bending performance

## Abstract

A co-curing resin system consisting of 9368 epoxy resin for prepreg and 6808 epoxy resin for resin transfer molding (RTM) was developed. A corresponding preparation method for a novel polymer composite bolted T-joint with internal skeleton and external skin was proposed based on the prepreg-RTM co-curing process, and novel T-joints were fabricated. A series of conventional configuration T-joints based on the RTM process and T-joints made of 2A12 aluminum alloy were prepared simultaneously. Bending performances were studied on these T-joints experimentally. The results indicate that 9368 epoxy resin and 6808 epoxy resin exhibit good compatibility in rheological and thermophysical properties. The novel T-joints prepared with the prepreg-RTM co-curing process show no obvious fiber local winding or resin-rich regions inside, and the interface quality between the internal skeleton and the external skin is excellent. The main failure modes of the novel T-joint under bending load include the separation of the skin and skeleton and the fracture along the thickness on the base panel; the skeleton carries the main bending load, but there is still load transfer between external skin and internal skeleton through their interface. The internal damages of the novel T-joint are highly consistent with surface damages observed visually, facilitating the detection and timely discovery of damages. The initial stiffness, damage initiation load, and ultimate load of the novel T-joint are 1.65 times, 5.89 times, and 3.45 times that of the conventional T-joint, respectively. When considering the influence of the density, the relative initial stiffness and relative ultimate load of the novel T-joint are 1.44 times and 2.07 times that of the aluminum alloy T-joint, respectively.

## 1. Introduction

**Figure 1:**
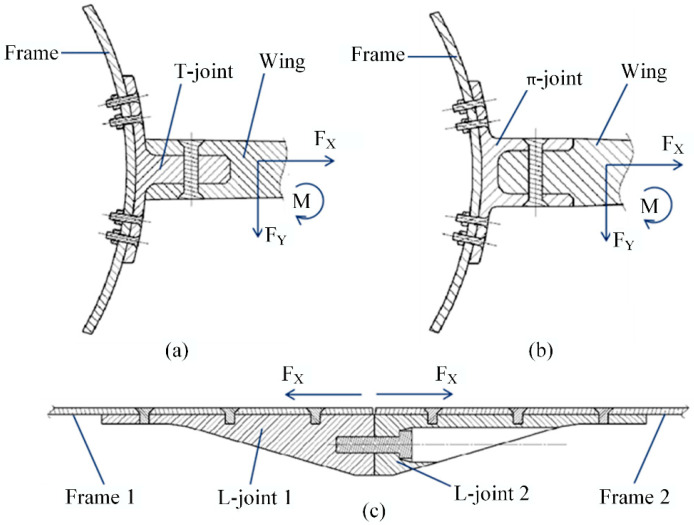
Connection joints mainly transmit out-of-plane loads, T-joint: (**a**), π-joint (**b**), and L-joint (**c**).

With the increasing demand for lightweighting in the aerospace field, advanced polymer composite structures have found increasingly extensive engineering applications [1,2]. Wherein, composite connection joints are utilized in secondary and primary load-carrying structures within wings, fuselages, wing-body junctions, etc. [1]. As for the connection forms, composite connection joints mainly include lap joints and insert (sleeve) joints used to transmit in-plane loads, as well as clevis and butt joints used for transmitting out-of-plane loads as depicted in Figure 1. Compared to the T-shaped longerons applied in fuselages and wings, the bolted T-joint, as shown in Figure 1a, features a thicker base panel and lug and is typically employed to transmit concentrated loads such as bending and tension between the wing and fuselage [3,4,5]. This not only leads to localized high stress regions in the corner area but also increases the design complexity of the composite T-joint.

**Figure 2:**
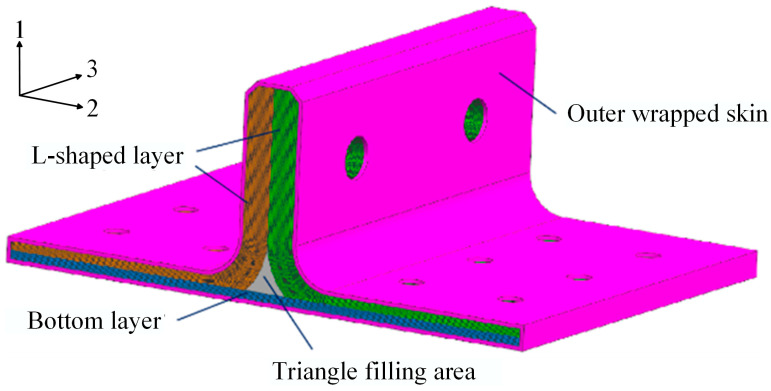
Conventional configuration of composite bolted T-joint.

As illustrated in Figure 2, the polymer composite bolted T-joint with conventional configuration (hereafter abbreviated as conventional T-joint) consists of two L-shaped layers, a bottom layer, a triangle filling area and outer wrapped skin, where L-shaped layers and base panel layers are composite laminates, and the triangle filling area mainly consists of resin matrix, short fibers, or twisted fibers [6]. When transverse bending loads are transmitted to the corner area of the T-joint, due to the significantly lower interlaminar mechanical properties of composite materials compared to that is in-plane [7], the conventional T-joint may experience failure modes such as bonding interface failure, laminate delamination, and random crack propagation within the triangle filling area [8]. In order to improve the load-carrying capacity of the conventional T-joint, some researchers have adopted methods such as stitching [9,10,11,12], Z-pin [13,14,15], and triangle filling area reinforcement [1,16,17] to delay the failure in interlaminar and triangle filling areas. Moderate stitching and Z-pins can effectively delay and reduce delamination damage to the joint and improve its load-carrying capacity [18], but they may also weaken the in-plane mechanical properties of laminates [19,20,21]. Moreover, the filling material in the triangle area is still connected to the L-shaped layer through the resin matrix, and the triangle filling area remains a weak position for bearing load, thus the degree of improvement in the mechanical performances of joints by this method is limited [17]. It can be seen that conventional T-joints have always struggled to address the problem of interlaminar load transfer in the corner area, severely limiting the application potential of composite T-joints.

**Figure 3:**
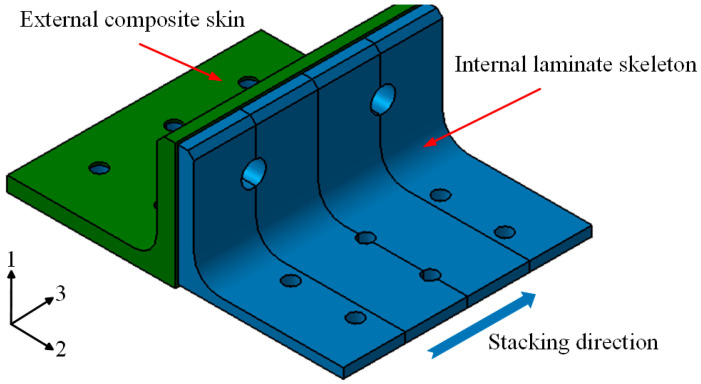
Novel configuration of composite bolted T-joint.

A polymer composite bolted T-joint with novel configuration (hereafter abbreviated as novel T-joint) consisting of an internal skeleton and external skin was proposed in reference [22], as shown in Figure 3. The novel T-joint can convert the interlaminar load in the corner area into the in-plane load of the skeleton, thereby significantly improving the bending performances of the joint. In order to prepare the novel T-joint with high bending performances, a prepreg-RTM co-curing process was adopted in reference [22]. The fundamental idea of this process involves using a prepreg layup to fabricate features such as bosses and stiffeners within the structure, overlaying large-area dry fabric skins on the prepreg skeleton, and then co-curing them through RTM process. Compared to the conventional autoclave process, this process can achieve net-size preparation of irregular contoured structures and integral stiffened panels without secondary bonding or mechanical connection methods, and compared to pure RTM process, this process does not require complex overall dry preform preparation and can improve defects such as local resin-rich areas and high porosity easily occurring in RTM process [23,24,25,26]. However, when using the prepreg-RTM co-curing process to prepare the T-joint, to ensure the quality of the interface between the skeleton and skin, the compatibility between the resin systems of the prepreg laminate and RTM is essential. If the thermophysical properties and rheological characteristics of the two resin systems are not matched, defects such as fiber wrinkling, delamination, and dense porosity are prone to occur at the interface [24,27,28,29]. Therefore, to produce the novel T-joint with high-quality interface using the prepreg-RTM co-curing process, it is necessary to address the compatibility of the co-curing resin system and the process adaptation issues.

Building on the novel configuration of composite T-joints proposed in reference [22] and the investigation on the bending failure mechanism for this novel T-joint, in order to achieve high-quality preparation of the novel T-joint based on the prepreg-RTM co-curing process, a co-curing resin system suitable for this process and a corresponding preparation method combining pre-compaction of prepreg and dry fabric RTM co-curing were developed here, and novel T-joints were fabricated. A series of conventional configuration T-joints based on the RTM process and T-joints made of 2A12 aluminum alloy were prepared simultaneously. Finally, the profile of the novel T-joint was inspected by CT scanning and optical imaging methods, and bending tests were conducted on the three types of T-joints to study the interface quality, failure modes, and bending performances of the polymer composite bolted T-joint based on the newly developed preparation method. The achievements of this study can provide references for the design and preparation of composite joints primarily subjected to out-of-plane loads.

## 2. Co-Curing Resin System Analysis

### 2.1. Test Methods

To determine the compatibility of the co-curing resin system, it is necessary to conduct rheological and thermophysical characterization tests on different resins. The properties to be tested and their corresponding methods and instruments used in this study are as follows:

(1) Glass transition temperature and Dynamic mechanical analysis (DMA): DMA is conducted using the Q800 DMA Dynamic Mechanical Analyzer from TA Instruments, DE, USA, following the ASTM D7028 standard [30].

(2) Viscosity-time relationship: Analysis of viscosity–time relationship is performed using the NDJ-7 Rotary Viscometer from Techcomp, Shanghai, China, following the GB/T 2794-2022 standard [31].

(3) Viscosity–temperature relationship: The viscosity–temperature relationship is analyzed using the HR20 Discovery Rheometer from TA Instruments, DE, USA following the GB/T 265-1988 standard [32].

(4) Gel time: The gel time of different resins are determined by conducting filament draw tests at different temperature points on a hot plate fixture, following the ASTM D3532 standard [33].

(5) Differential scanning calorimetry (DSC) analysis: DSC analysis is performed using the DSC-2910 Differential Scanning Calorimeter from TA Instruments, DE, USA, following the ISO 11357-2:2020 standard [34].

### 2.2. Co-Curing Resin System Design

To achieve compatibility in the co-curing resin system, the following requirements are imposed on the prepreg resin and the RTM resin. For the prepreg resin (1) little to no curing reaction occurs under storage conditions at room or lower temperature, and during the pre-compaction period at 90 °C and (2) maintain high viscosity characteristics during resin injection. For the RTM resin (1) possess chemical compatibility with the prepreg resin and (2) maintain low viscosity characteristics during resin injection.

Based on the requirements above, the prepreg adopts the resin designated as 9368, a mid-temperature curing epoxy resin system, with its main components being bisphenol A epoxy and latent curing agent dicyandiamide, with a mass ratio of 100:6 and a glass transition temperature of 161.65 °C. Under urea catalysis conditions, this resin exhibits nearly no curing reaction below 120 °C, meeting the process requirements for laminating at room temperature and pre-compaction at 90 °C. The corresponding reinforcement material is ZT7G-12K carbon fiber, with a nominal areal density of 135 g/m^2^ for the prepreg, a resin mass fraction of 37%, and a single-layer cured thickness of 0.125 mm.

**Figure 4:**
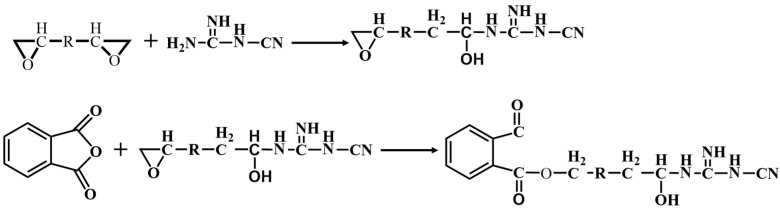
Reactions between epoxy resin, methyl tetrahydrophthalic anhydride, and dicyandiamide.

To ensure chemical compatibility between the RTM resin and the prepreg resin, the RTM resin also employs the bisphenol A epoxy as the fundamental component, along with methyl tetrahydrophthalic anhydride as the curing agent and tertiary amine as the accelerator, forming the main components of the RTM resin. As the anhydride curing agent exists in a low viscosity liquid state at room temperature, while the dicyandiamide curing agent in the prepreg resin is in granular form, the viscosity of the prepreg resin at the gel temperature is much higher than that of the RTM resin. This makes it difficult for the two resin systems to diffuse into each other during curing, with chemical reactions occurring only at the interface between the two resin systems. Furthermore, as shown in Figure 4, epoxy resin, methyl tetrahydrophthalic anhydride, and dicyandiamide all possess multiple functional groups, allowing epoxy molecules to serve as cross-linkers for the anhydride and dicyandiamide, facilitating curing and cross-linking at the interface between the two resin systems, thereby forming a continuous phase structure at the interface.

**Figure 5:**
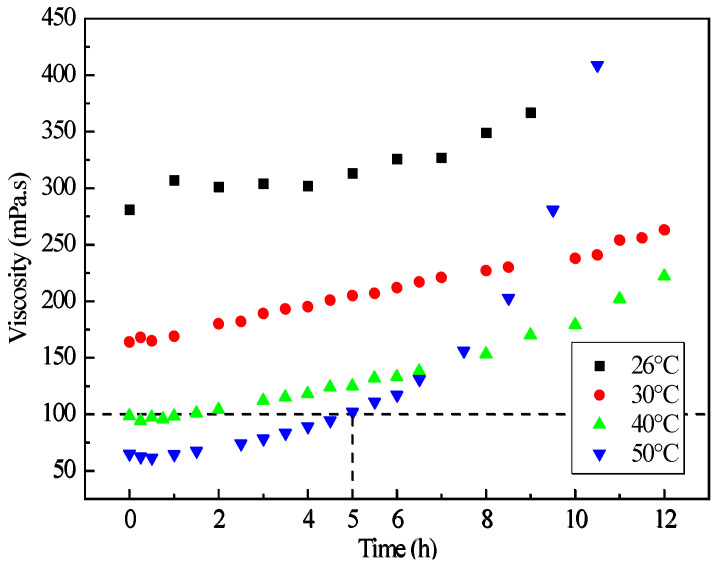
Relationship of viscosity and time of 6808 resin at different temperatures.

Based on the above main formulation design, the grade of the RTM resin system is designated as 6808, where bisphenol A epoxy, methyl tetrahydrophthalic anhydride curing agent, and tertiary amine accelerator are in a mass ratio of 50:50:1, with a glass transition temperature of 153.3 °C. As shown in Figure 5, the relationship of viscosity and time of 6808 resin system at different temperatures indicates that when the resin injection temperature is 50 °C, the RTM resin can maintain a viscosity of no more than 100 mPa·s for up to 5 h, meeting the requirements of low viscosity and long working period during the RTM injection process. The corresponding reinforcement materials include ZT7G-12K warp-knitting fabric, with an areal density of 135 g/m^2^ and a single-layer cured thickness of 0.125 mm, and G0814 plain weave fabric with fibers of grade ZT7G-3K, with a single-layer areal density of 200 g/m^2^ and a single-layer cured thickness of 0.2 mm. Additionally, the dry fabric shaping material used in the RTM process is Tack-328, a thermoplastic material with good compatibility with 6808 epoxy resin system.

### 2.3. Compatibility Analysis

**Figure 6:**
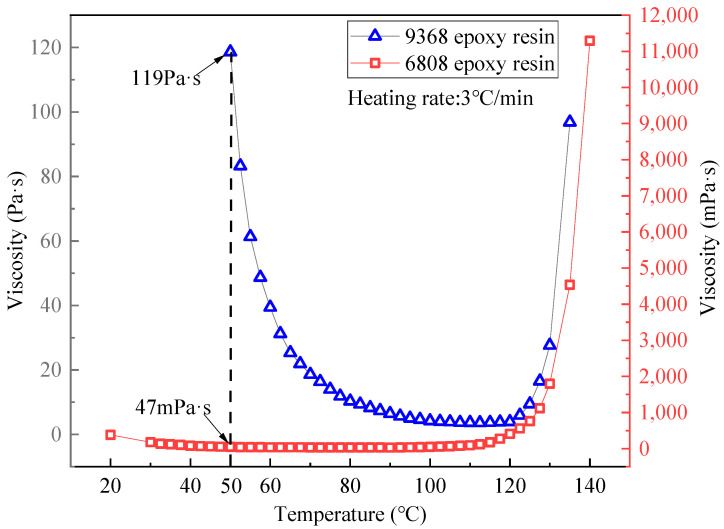
Viscosity–temperature curve of 9368 epoxy resin and 6808 epoxy resin.

**Figure 7:**
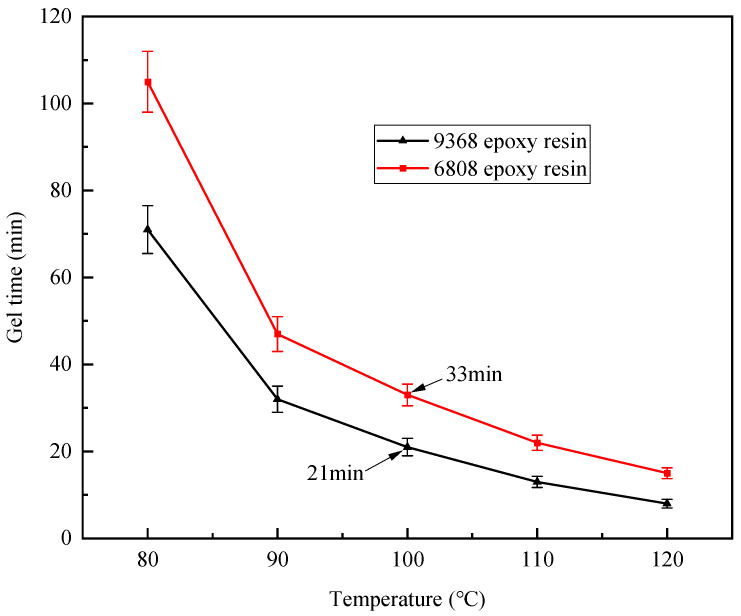
Gelation time curves of 9368 epoxy resin and 6808 epoxy resin.

As mentioned earlier, the compatibility between the prepreg resin and the RTM resin is crucial for achieving the prepreg-RTM co-curing process, where rheological and thermophysical properties serve as key indicators of compatibility. Figure 6 illustrates the viscosity–temperature curves of 9368 resin and 6808 resin. It is observed that when the 6808 resin is injected at 50 °C, the viscosity of the 9368 resin is approximately 119 Pa·s, while that of the 6808 resin is about 47 mPa·s, indicating a significant disparity in viscosity between the two resins. Consequently, there is minimal interdiffusion at the interface between the two resins, and the flow of the RTM resin has minimal impact on the prepreg resin. Additionally, the gelation time curves of 9368 resin and the 6808 resin are presented in Figure 7. The results indicate that the difference in gelation time between the two resins decreases with increasing temperature. Particularly, when the temperature exceeds 100 °C, the gelation time differs by only about 10 min, demonstrating comparable gelation time. Thus, 9368 resin and 6808 resin exhibit process compatibility in terms of rheological properties.

**Figure 8:**
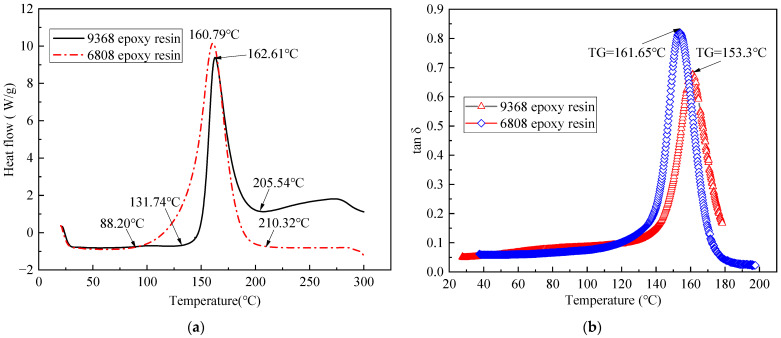
DSC curves (**a**) and DMA curves (**b**) of 9368 epoxy resin and 6808 epoxy resin.

DSC and DMA tests were conducted separately within the heating scans of 20~300 °C and 20~200 °C, yielding the DSC and DMA curves for the two resin systems as depicted in Figure 8. It is observed that both 9368 resin and 6808 resin exhibit a single main exothermic peak throughout the entire curing process, indicating a one-step reaction for curing, and their main exothermic peaks occur at nearly the same position. Moreover, 9368 resin and 6808 resin demonstrate similar glass transition temperatures of 161.65 °C and 153.3 °C, respectively, indicating good compatibility in terms of thermophysical properties.

In summary, 9368 epoxy resin and 6808 epoxy resin meet the compatibility requirements for the prepreg-RTM co-curing process, providing a material basis for the high-quality preparation of the novel polymer composite bolted T-joint with high-bending-performance.

## 3. Process of Novel Configuration T-Joint

### 3.1. Process Design

**Figure 9:**
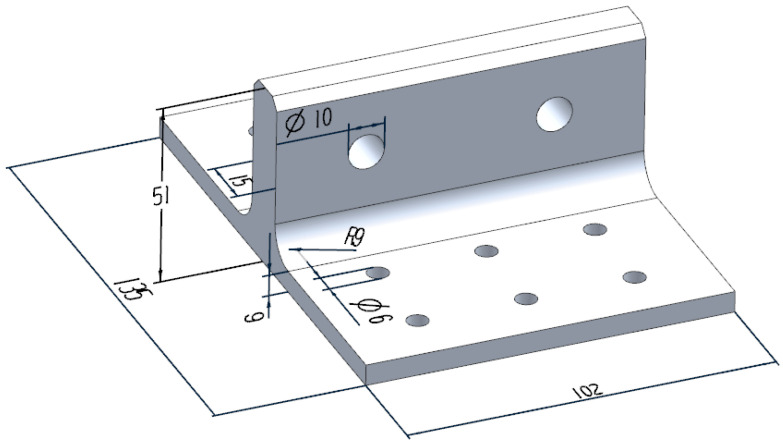
The dimension diagram of the novel T-joint. (The unit of length is all millimeters).

**Figure 10:**
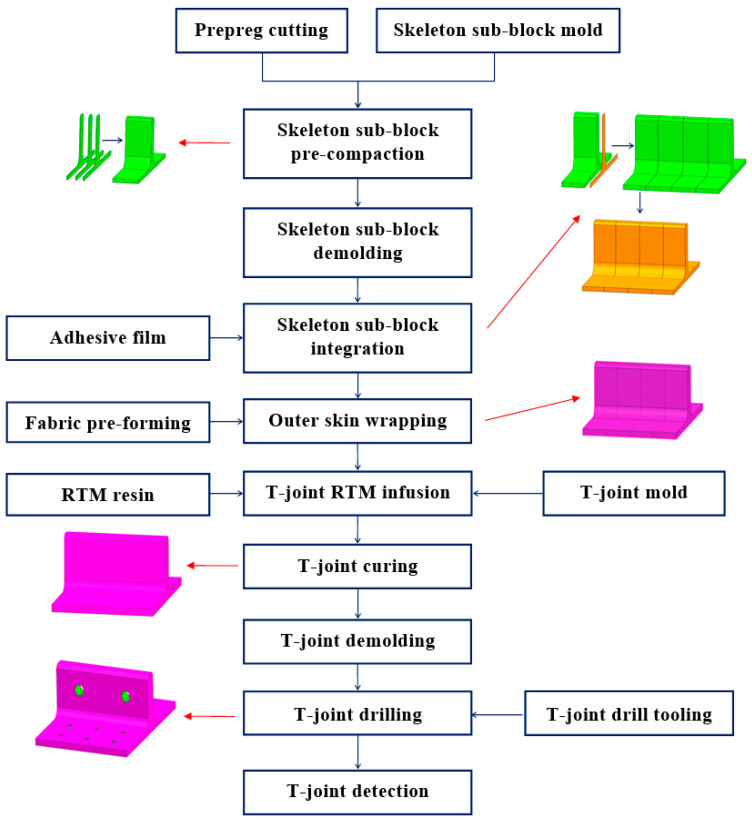
Prepreg-RTM co-curing process of the novel polymer composite bolted T-joint.

The geometric dimensions of the novel polymer composite bolted T-joint are illustrated in Figure 9. As depicted in Figure 3, the novel T-joint consists of internal skeleton and external skin. The internal skeleton consists of four sub-blocks, each formed by stacking prepreg layers along direction 3. The external skin is composed of fabrics, including woven and wrap-knitting fabrics, wrapped around the skeleton. Depending on the usage requirements, novel T-joints with different stacking sequences for the skeleton and skin can be designed. Based on the configuration of the novel T-joint and the selected co-curing resin system of the 9368 resin and 6808 resin, the prepreg-RTM co-curing process is designed as depicted in Figure 10. This process primarily includes: (1) prepreg cutting and sub-block mold preparation; (2) pre-compaction of the internal skeleton sub-blocks; (3) demolding of the sub-blocks; (4) bonding of the internal skeleton using adhesive film; (5) overall wrapping of the external skin with pre-formed fabric; (6) injection of RTM resin into the RTM mold; (7) co-curing; (8) demolding of the T-joint; (9) hole drilling; and (10) non-destructive testing of the T-joint.

### 3.2. RTM Injection Flow Analysis

**Figure 11:**
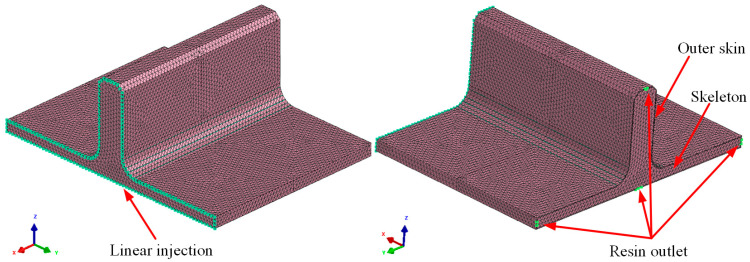
RTM injection flow simulation model.

In the RTM process, the curing time can account for up to 80% of the entire RTM process, and excessively long resin injection time can also increase the viscosity of the RTM resin [35]. Therefore, it is necessary to analyze the flowability of the RTM resin during the prepreg-RTM co-curing process. Based on the structural form of the novel T-joint, a design scheme with straight injection channels is adopted to simplify the mold structure and ensure overall sealing, with a linear injection inlet and four resin outlet points designed at both ends of the T-joint. The injection pressure is set to 0.6 MPa positive pressure at the inlet and −0.1 MPa vacuum negative pressure at the outlet. A simulation model was established using PAM-RTM 2019 commercial software to analyze the RTM resin injection process, as depicted in Figure 11. The mesh was generated using four-node tetrahedral elements, resulting in a total of 321,300 elements. The thickness of the outer skin dry fabric was 1 mm, with a fiber density set at 1.6 g/cm^3^, while the fiber volume fraction of internal skeleton was 60.9%, with a density of 1.55 g/cm^3^. The resin viscosity was 100 mPa·s, with a density of 1.2 g/cm^3^ and the permeability in the X, Y, and Z directions was set to 2 × 10^−13^ m^2^, where the X, Y, and Z directions correspond to direction 3, direction 2, and direction 1 in Figure 2, respectively.

**Figure 12:**
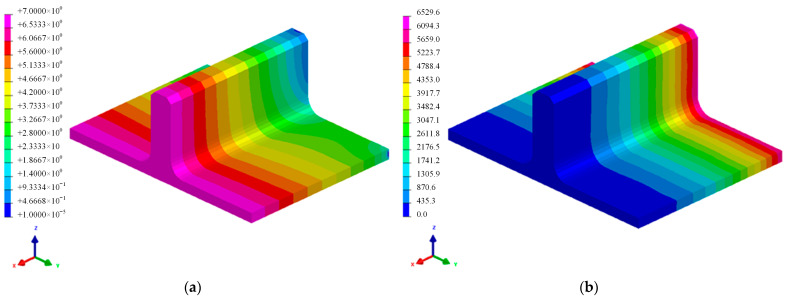
RTM injection flow analysis results: saturated seepage pressure distribution (**a**) and mold filling time (**b**).

The results of the RTM resin flow analysis, as shown in Figure 12, indicate that the distribution of saturated permeation pressure confirms the absence of dry spots during the injection process. Additionally, the mold filling time for injection is 6529.6 s, satisfying the process stability requirements of 6808 resin which can maintain a viscosity of no more than 100 mPa·s for up to 5 h at the injection temperature of 50 °C. Hence, this RTM resin injection scheme is suitable for the fabrication process of the novel T-joint proposed in this study.

### 3.3. Preparation Procedure

**Figure 13:**
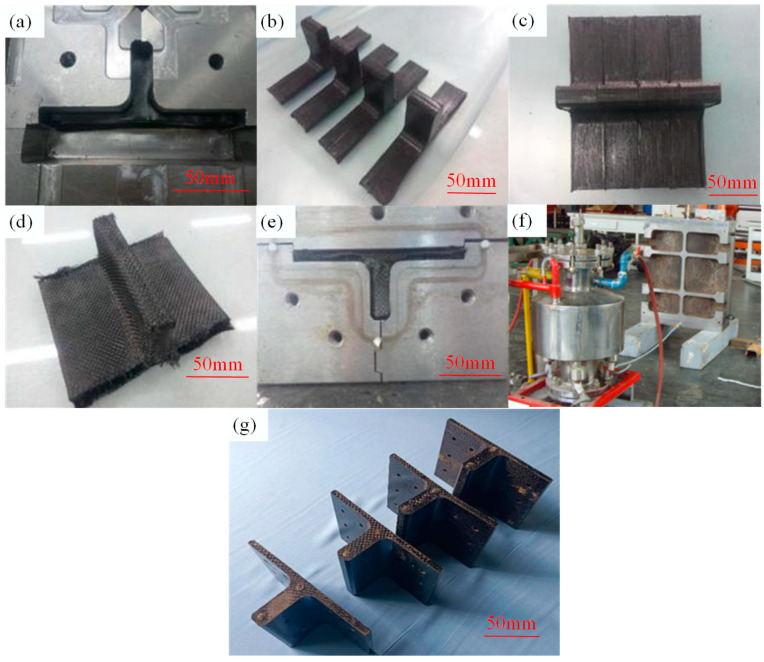
Preparation procedure of the novel polymer composite bolted T-joint: sub-block pre-compression (**a**), sub-blocks after pre-compression (**b**), sub-block integration (**c**), outer skin wrapping (**d**), mold Clamping (**e**), resin injection and co-curing (**f**), and prepared novel composite bolted T-joint (**g**).

According to the prepreg-RTM co-curing process depicted in Figure 10, the specific preparation procedure for the novel T-joint is illustrated in Figure 13, encompassing the following steps: (1) Using an automatic cutting machine, ZT7G/9368 prepreg layers are cut to predetermined geometric dimensions and laid up in the [0/+45/90/−45]_ns_ sequence into preforms of sub-blocks, where the 0° direction corresponds to direction 1 in Figure 3, and the 90° direction corresponds to direction 2 in Figure 3. Then, the sub-blocks are pre-compacted and molded using a compression molding method at 90 °C, with a dwell time of 30 min, followed by natural cooling under pressure to room temperature before demolding for subsequent use. (2) Four sub-blocks are bonded together using 9368 resin adhesive film to form the integral structure of skeleton. (3) As per the design requirements of stacking sequence of skin, skeleton was wrapped with ZT7G wrap-knitting fabric and ZT7G/G0814 plain weave fabric. (4) T-joint preforms are placed into the RTM co-curing mold, and after completing the sealing inspection, RTM resin injection is carried out at 50 °C, with injection pressure of 0.6 MPa at the inlet and −0.1 MPa vacuum pressure at the outlet. Then, the injection completion is confirmed by observing resin flow at the outlet. (5) After injection, the mold is placed in the oven and ramped up to 90 °C at a rate of 3 °C/min, held for 30 min, then further ramped up to 130 °C at the same rate and held for 120 min. (6) The mold is naturally cooled to room temperature before demolding, followed by hole drilling on the T-joint. (7) Non-destructive testing is conducted on the prepared novel polymer composite bolted T-joint to ensure quality assurance.

## 4. Experiment

### 4.1. Specimens

In order to evaluate the bending performances of the novel polymer composite bolted T-joint fabricated using the aforementioned prepreg-RTM co-curing process, six novel T-joints (N1~N6) with different skin sequences were prepared. Additionally, T-joints (C1, C2) with conventional configuration based on the RTM process and 2A12 aluminum alloy T-joints (AL1, AL2) were prepared to validate the superior bending performance of the novel T-joint. Among them, the conventional T-joints were laid up with [45/0/−45/90]_ns_ fabric layers along the L-region using ZT7G wrap-knitting fabric, with external wrapping of T300/G0814 plain weave fabric. The specific information of all test specimens is detailed in Table 1, where the 0° direction and 90° direction correspond to direction 3 and direction 2 in Figure 3, respectively.

### 4.2. Test Procedure

**Figure 14:**
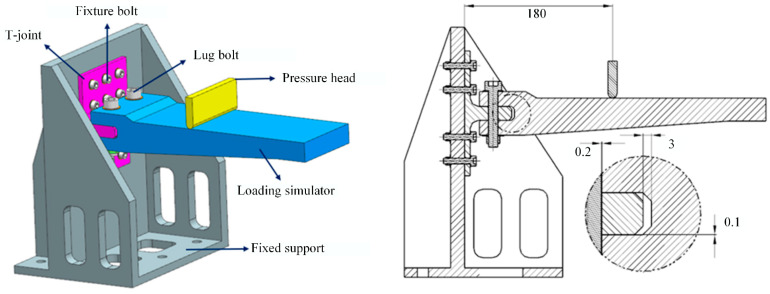
The diagram of loading scheme. (The unit of length is all millimeters).

**Figure 15:**
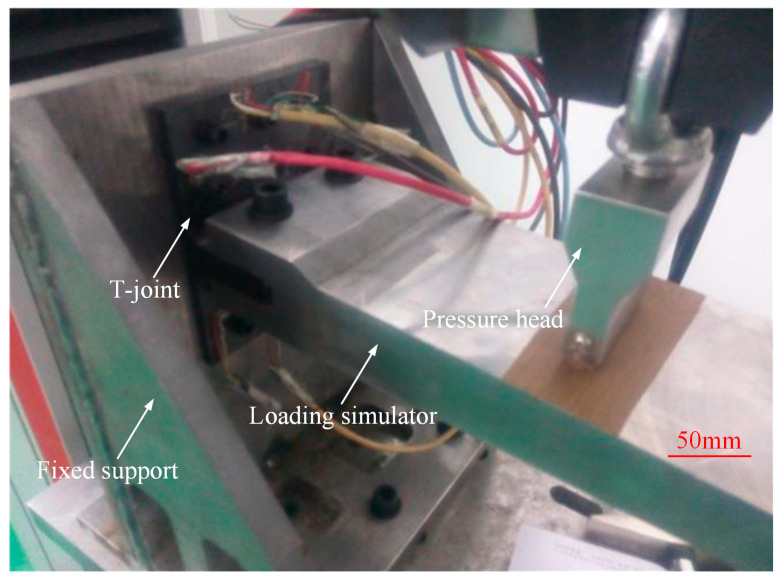
The experimental setup.

**Figure 16:**
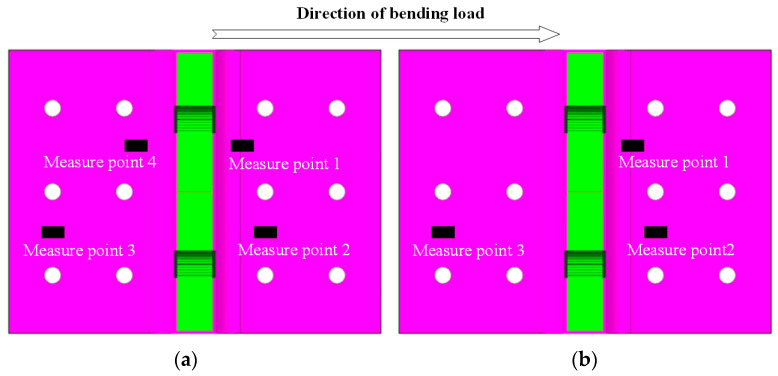
Arrangement of strain gauges: novel T-joint N2 (**a**) and conventional T-joint C1 (**b**).

The loading scheme and test setup are depicted in Figure 14 and Figure 15, respectively. The test fixture system comprises the wing loading simulator, pressure head, and fuselage simulation fixture (fixed support) used for loading and securing the T-joint, while according to the actual usage conditions of the joints, connecting fasteners were designed to form the test loading fixtures for the T-joint. The fixed support is fabricated from Q345 steel through welding and machining processes, with its bottom connected to the Instron 8801 testing machine. The wing loading simulator and pressure head are made of 45# steel. The connecting fasteners between the T-joint and the fixed support consist of 12 M6 high-strength steel bolts, while those between the T-joint and the wing loading simulator consist of 2 M10 high-strength steel bolts, which apply loads to the T-joint through the pressure head. According to an actual service condition of the T-joint in a certain engineering project, the length of the loading arm between the pressure head and the bottom of the T-joint base panel is set to 180 mm. Additionally, to avoid interference assembly between the lug of the T-joint and the wing loading simulator, assembly clearances of 0.1 mm and 0.2 mm are, respectively, provided between the lug and the simulator assembly surface, as well as between the two fastening bolts and the mating surface of the T-joint. The loading speed of the pressure head is set to 0.5 mm/min. During the test, displacement and load data are recorded by the testing machine. Furthermore, to compare the strain distribution at critical locations between the novel T-joint and conventional T-joint, strain tests were conducted on T-joints N2 and C1, with the arrangement of strain gauges illustrated in Figure 16.

## 5. Results and Discussion

### 5.1. Interface Quality

**Figure 17:**
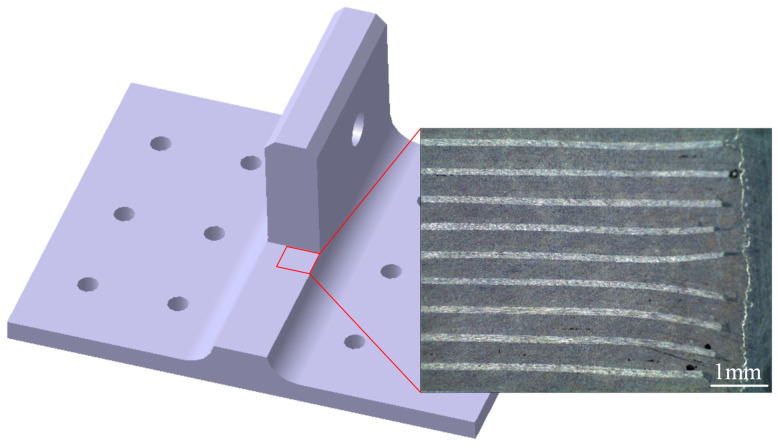
Profile optical image of the novel T-joint.

After curing, the internal quality of the novel T-joint was tested using optical imaging, as shown in Figure 17. The optical image indicates that the internal skeleton is well bonded with external skin, showing no visible delamination cracks or large-scale dense pores. However, there are still some small-sized void defects, primarily located near the junction between the prepreg layup and the RTM fabric layup. The possible cause of these defects lies in the inadequate precision of cutting the laminate prepreg during the layup process. In summary, it is evident that the novel T-joint utilizing 9368 resin and 6808 resin systems exhibits good interface quality between internal skeleton and external skin.

### 5.2. Failure Modes

**Figure 18:**
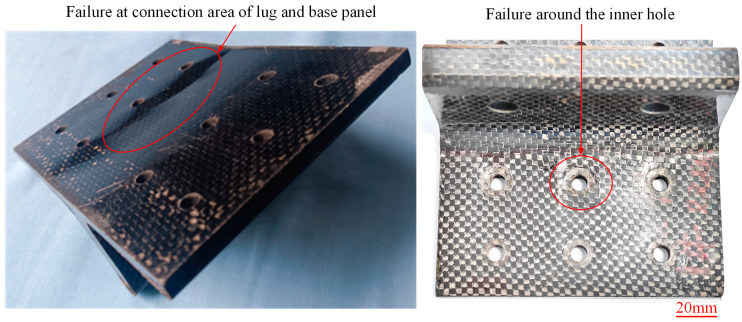
Visible typical failure modes of the novel T-joint. (The pictures were taken from specimens N5 (the left one) and N1 (the right one)).

**Figure 19:**
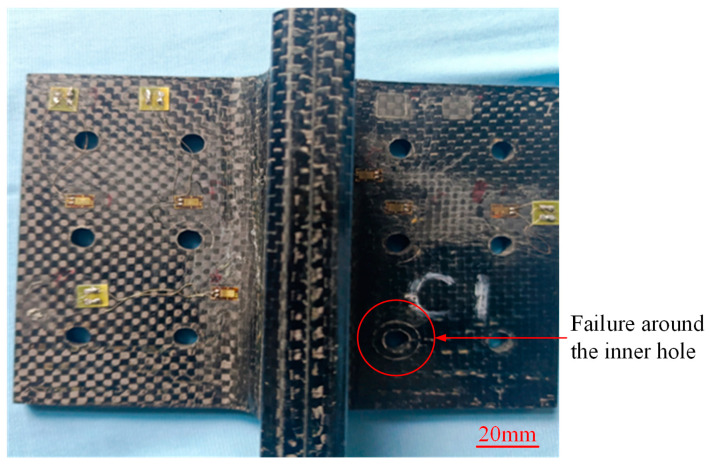
Visible typical failure modes of the conventional T-joint. (The pictures were taken from specimen C2).

The visualized typical failure modes of specimen N1~N6 after bending tests are depicted in Figure 18. All the novel T-joints exhibit bending fracture failures near the connection area of lug and base panel, with significant residual deformations and surface bulging observed, indicating the occurrence of delamination cracks between skin and skeleton. Additionally, noticeable compression failures are observed near the inner holes of the base panel, while there is almost no damage near the outer holes, suggesting that the inner holes bear greater loads, resulting in higher compression stress in this area and more significant damages. The visualized typical failure modes of the conventional T-joints are shown in Figure 19. Apart from slight compression traces around the inner holes, there are hardly any other visible damage modes observed.

**Figure 20:**
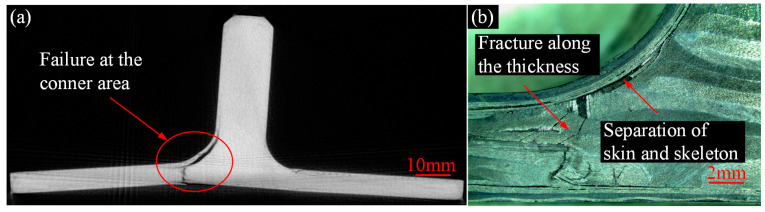
Results of the novel T-joint after experiment: CT scanning of specimen N5 (**a**) and profile optical imaging of specimen N4 (**b**).

**Figure 21:**
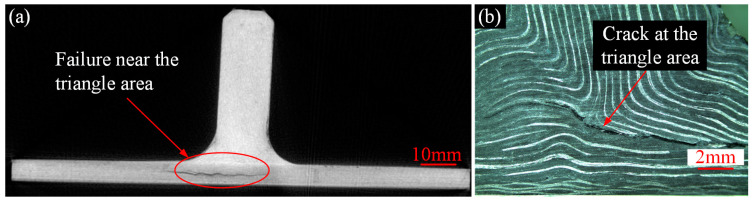
Results of the conventional T-joint after experiment: CT scanning of specimen C2 (**a**) and profile optical imaging specimen C1 (**b**).

To further investigate the failure behavior of composite T-joints with two configurations, CT scanning and optical imaging were conducted on specimen N6 and C1, yielding results as shown in Figure 20 and Figure 21. It is observed that the failure area of the novel T-joint concentrates in the corner region between lug and base panel, with significant separation between the skin and the skeleton, and the final fracture occurs along the thickness direction on the base panel. Due to the high fracture strength of the carbon fibers in the base panel, the energy absorbed during the fracture process is also high, resulting in a higher ultimate load-carrying capacity of the novel T-joint. In contrast, the final failure of the conventional T-joint manifests as a long crack in the triangle filling area. The main reason for the crack is the presence of severe fiber local winding and resin-rich areas in the triangle filling area, leading to poor mechanical performances, where damage can easily occur under relatively small loads, and continued loading leads to continuous crack propagation and eventual failure.

In summary, due to changes of the internal configuration, there is no obvious fiber local winding and resin-rich areas in the novel T-joint. According to the load distribution principle based on stiffness, the skeleton carries the main bending load, while the skin also carries a certain load apart from maintaining the overall shape of the joint. The final failure occurs in the base panel of skeleton, significantly enhancing the ultimate load-carrying capacity. Additionally, the comparison between visual and cross-sectional inspection results reveals a high degree of consistency between the internal damage and the surface damage, facilitating damage detection and timely identification.

### 5.3. Bending Performances

**Figure 22:**
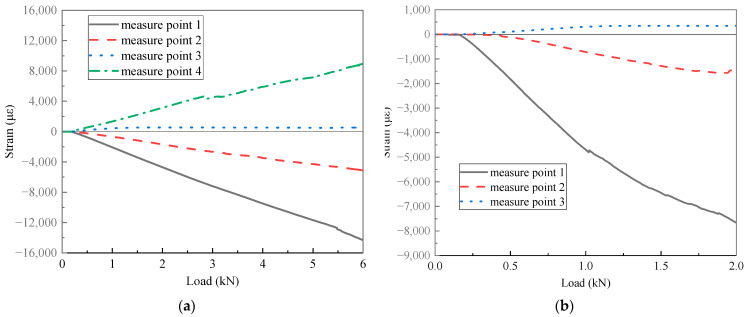
Strain–load curves of novel T-joint N2 (**a**) and conventional T-joint C1 (**b**).

The strain–load curves of specimens N2 and C1 are depicted in Figure 22, with the load of specimen N2 intercepted up to the damage initiation load of 6 kN, while the load of specimen C1 is intercepted up to a load of 2 kN in the linear segment of the load–displacement curve. It can be observed that the farther the strain gauge is from lug, the smaller the measured strain, confirming the viewpoint mentioned earlier that inner holes bear greater loads. Moreover, the strain of the novel T-joint exhibits higher linearity before the damage initiation load, indicating that significant internal damage has not occurred. Conversely, the conventional T-joint exhibits good linearity in the curve before 1 kN, but after 1 kN, it shows significant nonlinearity, indicating significant damage occurring within the joint near 1 kN. This also suggests that the novel T-joint possesses superior load-carrying capacity.

**Figure 23:**
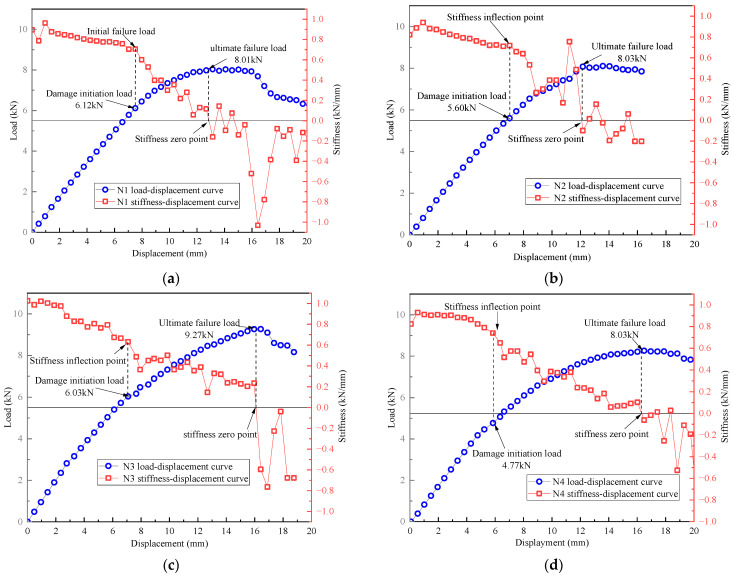

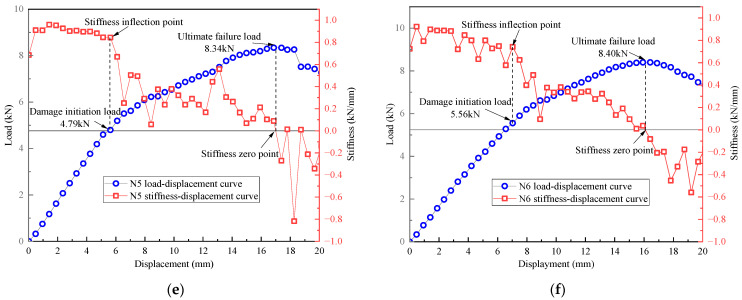
Load–displacement curves and stiffness–displacement curves of N1 (**a**), N2 (**b**), N3 (**c**), N4 (**d**), N5 (**e**), and N6 (**f**).

On the other hand, the load–displacement curves of the T-joints obtained from the tests, and the bending stiffness–displacement curves obtained by differentiating the bending load and displacement, are shown in Figure 23. It can be observed that the variation of bending stiffness of the novel T-joint can be roughly divided into three stages. In the initial stage, the bending stiffness of the novel T-joint remains relatively stable, with the bending load and displacement exhibiting good linearity. When load increases to about 6 kN, a turning point in bending stiffness occurs, with a noticeable decrease in the slope of the load–displacement curve, and the rate of increase in the load gradually decreases. When displacement is around 8 mm, the bending stiffness decreases to below 0.7 kN/mm. With further increase in load, the slope of load–displacement curve continues to decrease until reaching the ultimate load, with the ultimate load of ranging from 8.01 to 9.27 kN. With further increase in displacement, bending stiffness becomes negative, indicating loss of load-carrying capacity by the novel T-joint.

Analyzing the above changes in conjunction with the CT scanning results in Figure 20. It is observed that in the initial stage, both the internal skeleton and external skin bear bending loads simultaneously. However, as the load increases, the area of separation between skin and the skeleton and the internal damage area of the skeleton continue to increase, leading to a continuous decrease in the load-carrying capacity, resulting in a decrease in bending stiffness. With further increase in load, when the base panel of skeleton fractures, the T-joint loses its load-carrying capacity. Therefore, although the novel T-joint mainly bears bending loads through skeleton, there is still load transfer between skin and skeleton. Moreover, when the interface between skin and skeleton is damaged, the load-carrying capacity of skin will significantly decrease, thereby reducing the overall load-carrying capacity. Obviously, enhancing the interface performances between skin and skeleton could significantly improve the load-carrying capacity of the novel T-joint.

### 5.4. Comparison among Three Types of Joints

**Figure 24:**
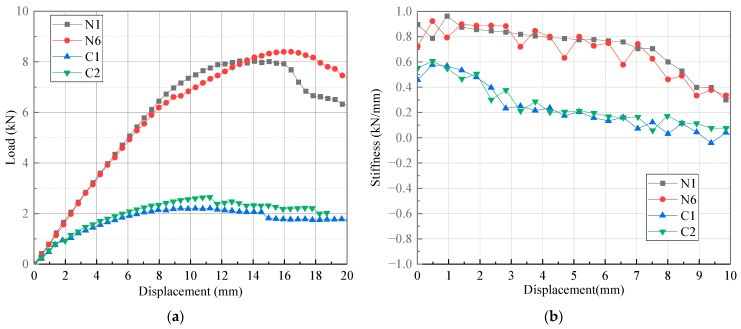
Load–displacement curves (**a**) and stiffness–displacement curves (**b**) of specimens N1, N6, C1, and C2.

**Figure 25:**
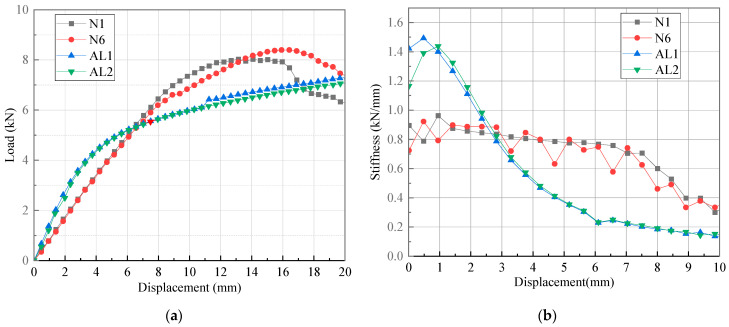
Load–displacement curves (**a**) and stiffness–displacement curves (**b**) of specimens N1, N6, AL1, and AL2.

The bending test results of the novel T-joint are compared with those of the conventional T-joint and 2A12 aluminum alloy T-joint to evaluate the excellence of the prepreg-RTM co-curing process, as shown in Figure 24 and Figure 25, Table 2, Table 3 and Table 4. Here, the initial stiffness is defined as the stiffness value corresponding to a displacement of 2 mm on the stiffness–displacement curve, the damage initiation load is defined as the load value corresponding to the inflection point on the stiffness–displacement curve, and the ultimate load of the composite T-joint is defined as the maximum load during the bending test, while for the aluminum T-joint, it is defined as the load when the displacement is 18 mm (10% of the length of bending loading arm).

Compared with the conventional T-joint, it is found that both the stiffness and ultimate load of the novel T-joint are much higher. Combining the data in Table 2, Table 3 and Table 4, the average initial stiffness, average damage initiation load, and average ultimate load of the novel T-joint are 165.46%, 589.25%, and 345.08% of those of the conventional T-joint, respectively. This further demonstrates that the novel T-joint proposed in this paper with in-plane load-carrying configuration can achieve a significant improvement in performance under the same size conditions.

Compared with 2A12 aluminum alloy T-joint, it is observed that the initial stiffness of the aluminum T-joint is larger. However, with the increase in load, the aluminum T-joint undergoes significant plastic deformation, leading to a rapid decrease in stiffness, while the novel T-joint exhibits relatively excellent and sustained stiffness performance. In terms of ultimate load, due to the significant plastic deformation of the aluminum T-joint, its ultimate load value is significantly lower than that of the novel T-joint. While considering the effect of density, the relative average initial stiffness and relative average ultimate load of the novel T-joint compared to the aluminum T-joint are 144.11% and 207.50%, respectively. Obviously, the novel T-joint possesses significant lightweight advantages.

### 5.5. Preparation Method Assessment

Based on the various analysis results mentioned above, the preparation effectiveness of the novel polymer composite bolted T-joint, fabricated with the 9368 and 6808 co-curing resin system and the corresponding prepreg-RTM co-curing process, can be evaluated from the following three aspects:

(1) For the co-cured interface quality, the 9368 and 6808 co-curing resin system exhibits good interface quality, which guarantees the integrity of the novel T-joint composed of the internal skeleton and external skin, as well as the effective load transfer between the skeleton and the skin.

(2) For the failure modes, under bending loads, compared to the conventional configuration, the final failure mode of the novel T-joint is the fracture of the base panel instead of the failure of the triangle filling area, thereby enhancing its load-carrying capacity.

(3) For bending performances, when considering the influence of overall density, compared to aluminum alloy T-joints of the same size, the relative average initial stiffness and relative average ultimate load of the novel T-joint are 144.11% and 207.50%, respectively, showcasing the excellent lightweight advantage of the novel T-joint.

In conclusion, the novel polymer composite bolted T-joint based on the prepreg-RTM co-curing process not only exhibits excellent bending performances but also has significant lightweight advantages, demonstrating significant potential as a substitute for the aluminum alloy T-joint. The proposed preparation method based on the prepreg-RTM co-curing process provides an innovative solution for the lightweight and high-load design of composite structures subjected to predominantly out-of-plane loads.

## 6. Conclusions

A co-curing resin system consisting of 9368 epoxy resin for prepreg and 6808 epoxy resin for RTM was developed. A corresponding preparation method of the novel polymer composite bolted T-joint with the internal skeleton and external skin was proposed based on the prepreg-RTM co-curing process. Subsequently, bending performances of the novel T-joint were studied experimentally, and compared with that of the corresponding conventional configuration T-joint and 2A12 aluminum alloy T-joint. Finally, the profile of the novel T-joint was inspected by CT scanning and optical imaging. The following conclusions can be drawn:

(1) The 6808 epoxy resin developed based on 9368 epoxy resin exhibits good compatibility with 9368 epoxy resin in rheological and thermophysical properties, meeting the requirements of the prepreg-RTM co-curing process.

(2) The novel T-joints with in-plane load-carrying configuration prepared by the prepreg-RTM co-curing process show no obvious fiber local winding or resin-rich regions inside, and the interface quality between the internal skeleton and external skin is excellent.

(3) The main failure modes of the novel T-joint include separation of skin and skeleton and fracture along the thickness on the base panel; the skeleton carries the main bending load, while the skin also shares a certain load apart from maintaining the overall shape of the T-joint, but there is still load transfer between the external skin and internal skeleton through their interface.

(4) The internal damages of the novel T-joint are highly consistent with surface damages observed visually, facilitating the detection and timely discovery of damages.

(5) The initial stiffness, damage initiation load, and ultimate load of the novel T-joint are 1.65 times, 5.89 times, and 3.45 times that of conventional T-joint, respectively. When considering the influence of density, the relative initial stiffness and relative ultimate load of the novel T-joint are 1.44 times and 2.07 times that of aluminum alloy T-joint, respectively.

## Figures and Tables

**Table 1:** Arrangement of test specimens.

Specimen	Number of Specimens	Density (g/cm^3^)	Triangle Filling Area	Stacking Sequence of the Skin
N1	1	1.55	\	[((0/90)*_f_*)_5_]
N2	1			[0_2_/±45/(0/90)*_f_*]
N3	1			[90_2_/±45/(0/90)*_f_*]
N4	1			[90_4_/(0/90)*_f_*]
N5	1			[(±45)_2_/(0/90)*_f_*]
N6	1			[0_4_/(0/90)*_f_*]
C1, C2	2	1.55	Twisted fibers	[((0/90)*_f_*)_5_]
AL1, AL2	2	2.7	\	\

**Table 2:** Comparison of initial stiffness of different types of T-joints.

Specimen	Novel T-Joint						Conventional T-Joint		Aluminum T-Joint	
	N1	N2	N3	N4	N5	N6	C1	C2	Al-1	Al-2
Initial stiffness (kN/mm)	0.85	0.87	0.99	0.91	0.95	0.89	0.56	0.54	1.07	1.12
Average value	0.91						0.55		1.10	
Relative value	82.73%						50.00%		100%	
Relative value with consideration of density *	144.11%						87.10%		100%	

* $\mathrm{Relative}\;\mathrm{value}\;\mathrm{with}\;\mathrm{consideration}\;\mathrm{of}\;\mathrm{density}=\frac{\mathrm{Relative}\;\mathrm{value}\;\times \;\mathrm{the}\;\mathrm{density}\;\mathrm{of}\;\mathrm{the}\;\mathrm{aluminum}\;\mathrm{T-joint}}{\mathrm{the}\;\mathrm{density}\;\mathrm{of}\;\mathrm{the}\;\mathrm{T-joint}\;\mathrm{to}\;\mathrm{be}\;\mathrm{calculated}}$.

**Table 3:** Comparison of damage initiation load of novel T-joints and conventional T-joints.

Specimen	Novel T-Joint						Conventional T-Joint	
	N1	N2	N3	N4	N5	N6	C1	C2
Damage initiation load (kN)	6.12	5.60	6.03	4.77	4.79	5.56	0.93	0.93
Average value	5.48						0.93	
Relative value	589.25%						100%	

**Table 4:** Comparison of Ultimate load of different types of T-joints.

Specimen	Novel T-Joint						Conventional T-Joint		Aluminum T-Joint	
	N1	N2	N3	N4	N5	N6	C1	C2	Al-1	Al-2
Ultimate load (kN)	8.01	8.03	9.27	8.03	8.34	8.40	2.20	2.64	7.11	6.90
Average value	8.35						2.42		7.01	
Relative value	119.12%						34.52%		100%	
Relative value with consideration of density *	207.50%						60.13%		100%	

* $\mathrm{Relative}\;\mathrm{value}\;\mathrm{with}\;\mathrm{consideration}\;\mathrm{of}\;\mathrm{density}=\frac{\mathrm{Relative}\;\mathrm{value}\;\times \;\mathrm{the}\;\mathrm{density}\;\mathrm{of}\;\mathrm{the}\;\mathrm{aluminum}\;\mathrm{T-joint}}{\mathrm{the}\;\mathrm{density}\;\mathrm{of}\;\mathrm{the}\;\mathrm{T-joint}\;\mathrm{to}\;\mathrm{be}\;\mathrm{calculated}}$.

## Data Availability

The original contributions presented in the study are included in the article, further inquiries can be directed to the corresponding author.
